# 3D Modeling: Insights into the Metabolic Reprogramming of Cholangiocarcinoma Cells

**DOI:** 10.3390/cells13181536

**Published:** 2024-09-13

**Authors:** Giorgia Ciufolini, Serena Zampieri, Simona Cesaroni, Valentina Pasquale, Marcella Bonanomi, Daniela Gaglio, Elena Sacco, Marco Vanoni, Mirella Pastore, Fabio Marra, Daniel Oscar Cicero, Chiara Raggi, Greta Petrella

**Affiliations:** 1 Department of Chemical Science and Technology, University of Rome “Tor Vergata”, 00133 Rome, Italy; ciufolini@scienze.uniroma2.it (G.C.); serena.zampieri@students.uniroma2.eu (S.Z.); simona.cesaroni@alumni.uniroma2.eu (S.C.); cicero@scienze.uniroma2.it (D.O.C.); 2 Department of Biotechnology and Biosciences, University of Milan-Bicocca, 20126 Milan, Italy; valentina.pasquale@unimib.it (V.P.); elena.sacco@unimib.it (E.S.); marco.vanoni@unimib.it (M.V.); 3 SYSBIO-ISBE-IT-Candidate National Node of Italy for ISBE, Research Infrastructure for Systems Biology Europe, 20126 Milan, Italy; marcella.bonanomi@cnr.it (M.B.); daniela.gaglio@cnr.it (D.G.); 4 Institute of Bioimaging and Complex Biological Systems (IBSBC), 20054 Segrate, Italy; 5 Department of Experimental and Clinical Medicine, University of Florence, 50121 Florence, Italy; mirella.pastore@unifi.it (M.P.); fabio.marra@unifi.it (F.M.)

**Keywords:** cancer cell metabolism, metabolomics, NMR, LC-MS, spheroids, cholangiocarcinoma

## Abstract

Developing accurate in vitro models that replicate the in vivo tumor environment is essential for advancing cancer research and therapeutic development. Traditional 2D cell cultures often fail to capture the complex structural and functional heterogeneity of tumors, limiting the translational relevance of findings. In contrast, 3D culture systems, such as spheroids, provide a more physiologically relevant context by replicating key aspects of the tumor microenvironment. This study aimed to compare the metabolism of three intrahepatic cholangiocarcinoma cell lines in 2D and 3D cultures to identify metabolic shifts associated with spheroid formation. Cells were cultured in 2D on adhesion plates and in 3D using ultra-low attachment plates. Metabolic exchange rates were measured using NMR, and intracellular metabolites were analyzed using LC-MS. Significant metabolic differences were observed between 2D and 3D cultures, with notable changes in central carbon and glutathione metabolism in 3D spheroids. The results suggest that 3D cultures, which more closely mimic the in vivo environment, may offer a more accurate platform for cancer research and drug testing.

## 1. Introduction

Developing accurate in vitro models closely mimicking in vivo tumor environments is crucial for advancing cancer research and therapeutic development. Traditional 2D cell cultures, where cells are grown in flat, uniform layers, often fail to replicate the complex structural and functional heterogeneity of tumors in the body [1]. This limitation significantly impacts the translational relevance of findings from such models. In contrast, 3D culture systems, such as spheroids or organoids, offer a more physiologically relevant context by replicating key aspects of the tumor microenvironment, including cell–cell communication, oxygen and nutrient gradients, and waste accumulation [2]. Consequently, they provide a more accurate tool for studying tumor biology, drug penetration, and cellular responses to therapies, ultimately leading to more reliable preclinical testing phases and potentially enhancing the predictive accuracy of therapeutic efficacy and toxicity in clinical trials [3].

The transition from 2D to 3D cultures has revealed profound modifications in metabolic pathways [4], which is crucial for understanding the unique demands of tumor survival and proliferation. For instance, 3D tumor models frequently exhibit altered responses to pharmacological agents, suggesting enhanced chemoresistance often observed in clinical settings [5]. This shift has substantial implications for drug development, indicating that 3D cultures could lead to more predictive models for therapeutic efficacy.

Moreover, 3D models facilitate the study of the tumor microenvironment’s impact on cancer metabolism, providing insights into hypoxia-induced metabolic reprogramming, nutrient supply, and byproduct accumulation [6]. Understanding these interactions in 3D cultures enhances our knowledge of cancer biology, aiding in identifying novel metabolic targets and improving the specificity and effectiveness of anticancer therapies.

However, despite their utility, 3D cultures present several limitations that must be considered. Tumors are inherently heterogeneous, yet 3D models may not fully capture this complexity, leading to potential discrepancies in metabolic profiles [7]. Moreover, developing nutrient and oxygen gradients within 3D cultures can impact cellular behavior and metabolism, deviating from the uniform distribution observed in vivo. Scaling up 3D cultures for high-throughput applications poses challenges, and accessing the interior of spheroids for metabolic analysis can be technically demanding [8].

Despite the significant number of research studies comparing the two culture systems, open questions still need to be addressed to fully comprehend the utility of spheroids as tumor surrogates [9]. One possible strategy involves studying nutrient exchanges and internal pools simultaneously in 2D and 3D culture models. The relationship between nutrient exchange rates and internal pools is critical in cell metabolism, influencing cellular functions and survival strategies, particularly in tumor cells.

Nutrient exchange rates refer to the movement and utilization of nutrients within cellular environments, which are essential for supporting various metabolic activities. In tumor cells, these fluxes are influenced by the microenvironment, with distinct nutrient availability compared to normal tissues. Tumor cells adapt to these conditions through altered metabolic processes, utilizing available nutrients to support growth and proliferation [10,11]. Internal pools refer to the reservoirs of metabolites and nutrients within cells used for various biochemical reactions. In tumor cells, these pools are often dysregulated, leading to altered metabolic processes. For instance, tumor cells may upregulate arginine biosynthesis to cope with nutrient stress, highlighting the significance of understanding the dynamics between nutrient fluxes and internal pools [12].

Our study focused on three intrahepatic cholangiocarcinoma (iCCA) cell lines, CCLP1, HUCCT1, and SG231, cultured as 2D monolayers and 3D spheroids. In a previous paper, CCLP1 and HUCCT1 were used to explore the metabolic regulation of cancer stem cells (CSCs) in CCA [13]. The study found that CSCs in iCCA have a more efficient respiratory phenotype driven by mitochondrial oxidative phosphorylation, highlighting the pivotal role of mitochondrial metabolism in maintaining the CSC phenotype in iCCA. To this end, the stem-like subset was enriched by a 3D sphere culture containing a stem medium compared to parental cells grown as 2D monolayers.

Our study compared the metabolites of three iCCA cell lines in 2D and 3D cultures. We used NMR to measure nutrient exchange rates and LC-MS to determine metabolite abundance inside the cells. We aimed to identify metabolic shifts when moving from 2D to 3D cultures. We also explored the differences between the monolayers and spheroids in cell populations that contribute to the observed metabolic profiles.

## 2. Materials and Methods

The experimental section is described in detail in the Supplementary Materials document.

### 2.1. Cell Culture

The cholangiocarcinoma cell lines CCLP1, SG231, and HUCCT1 were routinely maintained as briefly indicated in the Supplementary Materials. For metabolomics experiments, cell lines were grown as monolayers (2D cultures) and spheroids (3D cultures) using the same medium (3D experimental medium; details in the Supplementary Materials). Sphere formation and cell adhesion were monitored through imaging and cell counting to assess morphology and viability, and medium and pellet samples were collected for analysis. Two biological replicates were conducted for each experiment, along with blank samples stored for NMR analysis at −80 °C.

### 2.2. Metabolic Data Acquisition

Media samples were thawed and deproteinized using ultrafiltration and mixed with phosphate buffer containing D_2_O, NaN_3_, and 3-(trimethylsilyl) propionic-2,2,3,3 acid sodium salt TSP-d_4_ as an internal standard and imidazole to check the pH. ^1^H-NMR spectra were acquired using a Bruker Avance 700 MHz NMR spectrometer (Bruker, Milan, Italy). Thirty-three metabolites were quantified using Chenomx NMR Suite 8.5 (Edmonton, AB, Canada), and the exchange rates were calculated using metabolite variations and cell growth curves, yielding consumption and excretion rates.

After an extraction procedure, the pellet metabolites were separated using Agilent 1290 UHPLC (Agilent Technologies, Milan, Italy) and detected using Agilent 6550 iFunnel Q-TOF MS (Agilent Technologies, Milan, Italy). The data were processed with MassHunter Profinder, and metabolites were identified with an in-house database. Peak areas were normalized for protein content.

### 2.3. Data Analysis

All data were normalized using probabilistic quotients [14] and Unit-Variation-scaled [15] to account for the different cell numbers or cell sizes between samples. Unsupervised Principal Component Analyses (PCA) and supervised Orthogonal Partial Least Squares Discriminant Analyses (OPLS-DA) [16] were conducted using SIMCA software (version 17, Umetrics, Sartorius Stedim Biotech, Gottingen, Germany). Variable importance for the Projection (VIP) was used to identify the metabolites mainly responsible for the separation. Additionally, ANOVA of the cross-validated residual (CV ANOVA) tests were performed to evaluate the significance of OPLS-DAs using SIMCA.

## 3. Results

### 3.1. Combining Exo- and Endometabolimics Data

We have compared the metabolism of three iCCA cell lines, CCLP1, HUCCT1, and SG231, cultured in 2D monolayers and 3D spheroids. To obtain a more comprehensive insight into cell metabolism, we combined two metabolomics approaches: endo- and exometabolomics. The former measures the intracellular metabolic pool at a given time in the culture, normalized by total protein content. In contrast, exometabolomics measures the metabolic exchange rates of cells with the culture medium over a period, expressed as pmol cell^−1^ day^−1^. The analytical platforms used to this end were LC-MS and NMR, respectively.

For each cell line and culture type, two biological replicates, each consisting of three technical replicates, were carried out. Representative images of the 2D and 3D cultures used for the metabolomic analysis are shown in Figure S1 (Supplementary Material). In the 2D experiment, exometabolomics analysis was performed on the medium, while endometabolomics analysis was conducted on the cell pellet after two days of culture. The same data were collected after five days of culture for the three-dimensional experiment. Cell-free blanks were also taken with the same culture conditions for each collection time, and cell counts were performed on days 0 and 2 for 2D and on days 0 and 5 for 3D.

**Figure 1:**
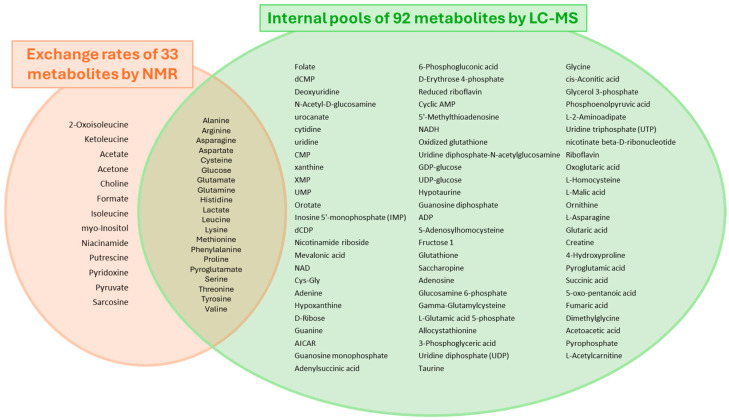
The cellular metabolome obtained for the three CCA cell lines combining exo- and endometabolomics.

Using this strategy, we collected data on thirty-three metabolites via NMR and ninety-two metabolites via LC-MS in both culture modes. Information about twenty metabolites resulted in common in the two datasets, which allowed us to explore the correlation between exchange rates and internal pools (Figure 1).

### 3.2. Cell Line Characteristics Mainly Influence Metabolic Fluxes, Whereas Culture Conditions Impact Internal Pools

**Figure 2:**
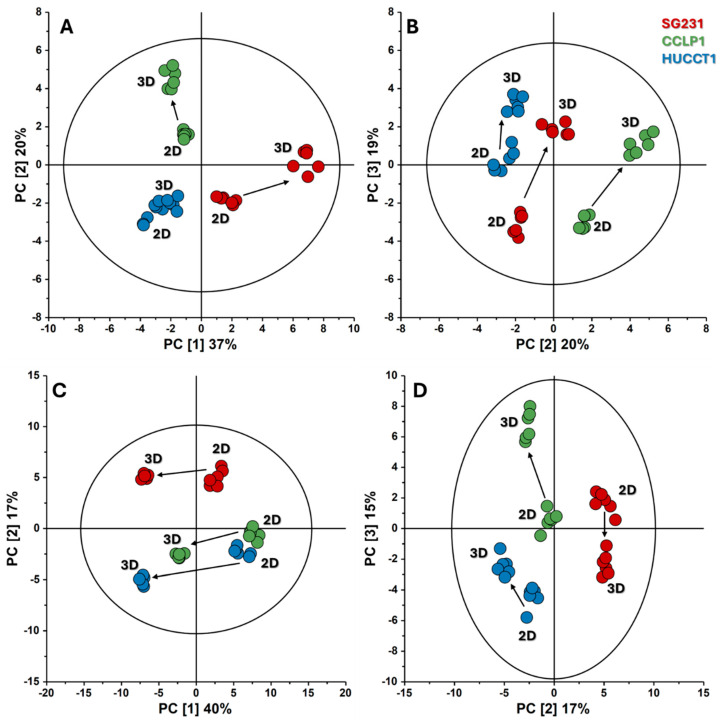
PCA score plots based on the metabolite exchange rates (**A**,**B**) and internal pools (**C**,**D**) of 2D and 3D cultures of CCA cell lines. The first three principal components were considered to achieve 76% and 72% of the total variance explained for the exchange rates and internal pools, respectively. Black arrows indicate the direction in which each cell line’s metabolism shifts from the 2D to the 3D culture. The figures were plotted using SIMCA-17.0.1 (Umetrics, Sartorius Stedim Biotech).

The NMR analysis of the metabolite concentrations in the culture medium resulted in the measurement of 38 exchange rates, as described in Supplementary Material. These variables were subjected to PCA, revealing that the three cell lines exhibited different profiles when transitioning from monolayers to spheroids (Figure 2A,B). The best model was obtained by considering the first three components, as the variance explained by the second (20%) and third (19%) components was only slightly different. When all three elements were considered, they accounted for 76% of the variance. The first and second principal components (PC [1] and PC [2]) discriminate the three cell lines. The difference between 2D and 3D culture types is evidenced only by the third component, PC [3]. This result suggests that the cell type is more significant in determining the exchange profile than the culture type.

The same unsupervised analysis was performed using the relative abundances within the cells in the two conditions obtained by LC-MS. They constitute a significant dataset of 92 variables and were used as the input for a PCA model (Figure 2C,D). Similar considerations as before led us to include the first three principal components, which account for 72% of the total variance. According to this model, PC [1] distinguishes the metabolite profiles of monolayers from those of spheroids. This suggests that the primary factor responsible for variations in the internal pools of these cell lines is their respective culture modes. This contrasts the role of PC [1] in the PCA model based on exchange rates (Figure 2A), which was to discriminate among the cell lines. This differentiation is carried out in the internal pool model by PC [2] and PC [3].

### 3.3. Pyruvate and Glutathione Metabolism Are Involved in the Monolayer-to-Spheroid Transition

**Figure 3:**
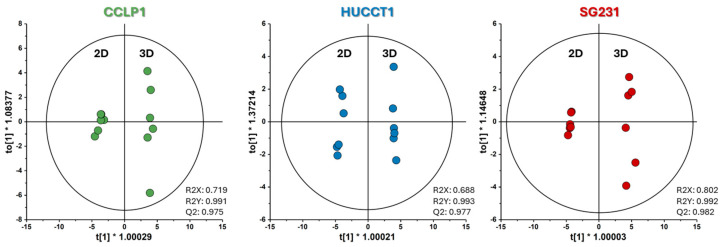
OPLS-DA score plots based on exchange rates of 2D vs. 3D of the three CCA cell lines. All models were validated using CV-ANOVA and permutation tests. The figure was plotted using SIMCA-17.0.1 (Umetrics, Sartorius Stedim Biotech).

To better determine the profile differences between 2D cultures and 3D spheroids, pairwise orthogonal partial-least square discriminant analysis (OPLS-DA) models were constructed on the exchange rates obtained for all cell lines (Figure 3).

**Figure 4:**
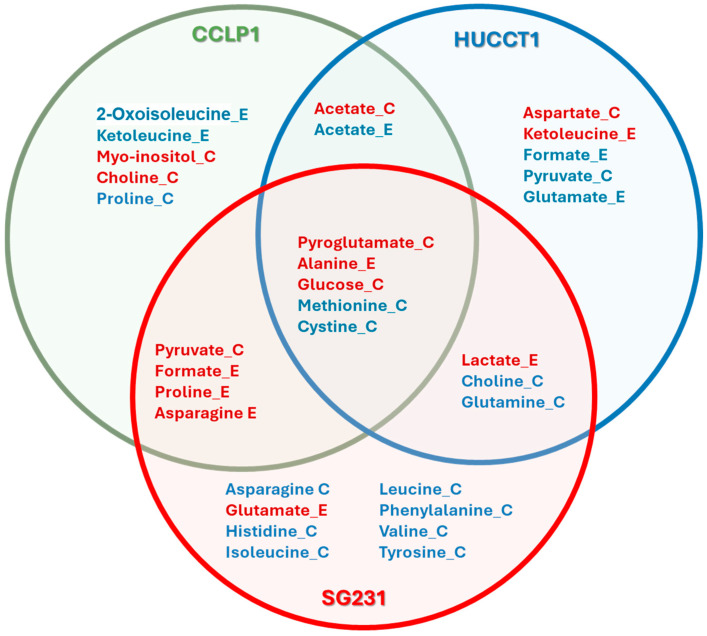
Metabolite exchange rates that discriminate between 2D and 3D culture profiles according to VIP classification, divided by cell line and culture model. C and E refer to consumption and excretion, respectively. The red and blue colors of metabolite names denote an increase or decrease in the respective exchange rates in the 3D culture model with respect to 2D.

The variables of importance in projection (VIP) were analyzed to determine the discriminatory exchange rates in the three cases. Those metabolites showing exchange rates with VIP > 1 were considered and clustered as a function of the cell line (Figure 4).

Independently of the cell lines, we observed an increase in the consumption of glucose and pyroglutamate and the excretion of alanine. On the contrary, the consumption of sulfur-bearing amino acids like methionine and cystine was diminished in spheroids with respect to monolayers. These five metabolites can be classified as part of the pyruvate metabolism (glucose and alanine) and the glutathione metabolism (pyroglutamate, cystine, and methionine). Pyruvate, which belongs to the first pathway, is consumed more by CCLP1 and SG231 but is decreased in HUCCT1 spheroids. Lactate, also part of the pyruvate metabolism, is excreted more by HUCCT1 and SG231 cells. Glutamate is also part of the glutathione metabolism, and its excretion is altered differently among the three cell lines: it was increased for SG231, decreased for HUCCT1, and unchanged for CCLP1. Glutamate, together with glutamine, intervenes in many pathways, and in particular, they take part in the alanine, aspartate, and glutamate metabolism, as well as the glyoxylate and dicarboxylate metabolism. As part of the first, aspartate and asparagine show different alterations in the transition between 2D and 3D cultures, as well as proline and histidine, both linked to glutamate. Glyoxylate and dicarboxylate metabolism play crucial roles in cellular metabolism, primarily in the metabolism of fats and carbohydrates. The metabolites found altered in this analysis, like acetate, pyruvate, and formate, belong to these pathways. Branched-chain amino acid (BCAA) metabolism also changes differently among the three lines in the transition between the two culture modes. Metabolites arising from BCAA breakdowns, like 2-oxoisoleucine and ketoleucine, are less excreted by CCLP1 spheroids, whereas the former shows higher excretion in HUCCT1 cells 3D cultures. The three BCAAs, isoleucine, leucine, valine, and other essential amino acids like histidine and phenylalanine, together with the correlated tyrosine, are consumed less by SG231 spheroids. The list is completed by two metabolites related to lipid metabolism: choline and myo-inositol.

**Figure 5:**
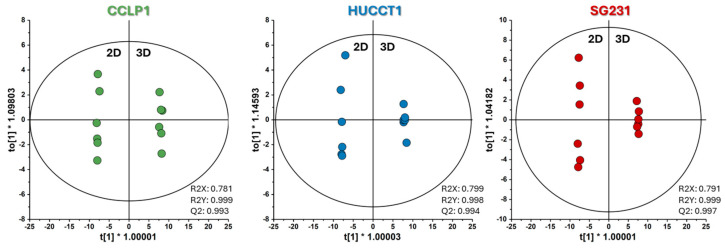
OPLS-DA score plots based on the three CCA cell lines’ metabolite internal pools of 2D vs. 3D culture modes. All models were validated using CV-ANOVA and permutation tests. The figure was plotted using SIMCA-17.0.1 (Umetrics, Sartorius Stedim Biotech).

We repeated the OPLS-DA analyses using the LC-MS data (Figure 5) to extract the metabolite levels most contributing to the changes between 2D and 3D cultures.

**Figure 6:**
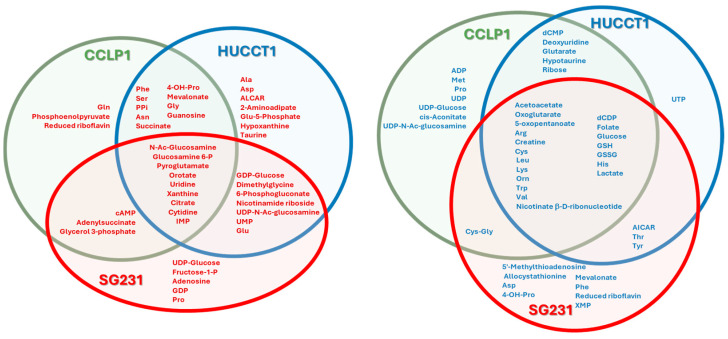
Metabolite internal pools that discriminate between 2D and 3D culture profiles according to VIP classification, divided by the cell line and culture model. The red and blue colors of metabolite names denote an increase or decrease in the respective internal pools in the 3D culture model with respect to 2D.

Significant separations and highly predictive models were obtained in all cases. The VIP score analysis allowed for the extraction of variations in all cases, as shown in Figure 6.

In the transition from monolayers to spheroids, twenty-eight metabolite internal pools were found to be altered in all cell lines. The most affected pathways were glutathione metabolism (glutathione, oxidized glutathione, cysteine, ornithine, and pyroglutamate), pyrimidine metabolism (cytidine, dCDP, orotate, and uridine), arginine and proline metabolisms (arginine, ornithine, oxoglutarate, and creatine), and the alanine, aspartate, and glutamate metabolism (citrate, glutamine-6-phosphate, and oxoglutarate). Furthermore, there was a significant decrease in the concentration of essential amino acids inside cells in spheroids, including leucine, valine, lysine, histidine, and tryptophan. It is worth noting that glucose and lactate internal pools were also decreased in spheroids, indicating a change in glycolysis regulation and pyruvate metabolism. When extending the analysis to metabolite pools altered in two cell lines, other pathways emerged, like glycine, serine, and threonine (creatine, cysteine, serine, glycine, dimethylglycine, and threonine) and the glyoxylate and dicarboxylate metabolism (citrate, serine, glycine, glutamate).

### 3.4. The Glutathione and Pyruvate Metabolism Alterations in Spheroids: Insights from Endo- and Exometabolomics Correlations

**Figure 7:**
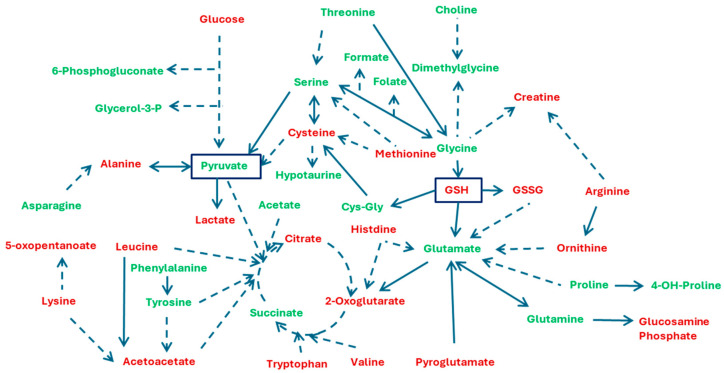
Metabolites whose exchange rates or internal levels were altered in 3D compared to 2D culture modes. Pyruvate and glutathione are highlighted due to their central role in linking all the metabolites. Red names refer to alteration in all three cell lines, whereas green names refer to alteration in two cell lines.

This study’s results demonstrate significant differences in metabolic pathways when comparing the exo- and endometabolomics profiles of monolayers and spheroids. We identified five exchange rates and twenty-eight internal metabolite concentrations that were significantly different in all cell lines between the two culture modes. Most metabolites belong to the central carbon metabolism. By adding the variables altered in at least two cell lines, we can create a highly interconnected network of pathways that illustrates how cellular metabolism behaves differently in 2D vs. 3D cultures (Figure 7).

Enrichment analysis using MetaboAnalyst v. 6.0, an online software tool [17], revealed that the main pathways resulting from this set of metabolites include alanine, aspartate, and glutamate metabolism, glutathione metabolism, arginine biosynthesis, and glyoxylate and dicarboxylate metabolism. The scheme presented in Figure 7 highlights the central roles played by pyruvate and glutathione as intersections of these interconnected pathways.

To better understand the correlation between these two datasets, we have created a single graph that accounts for both nutrient exchange rates and internal pools. This graph provides an integrated view of how external nutrients are processed and utilized within the cell.

#### 3.4.1. Pyruvate Metabolism

**Figure 8:**
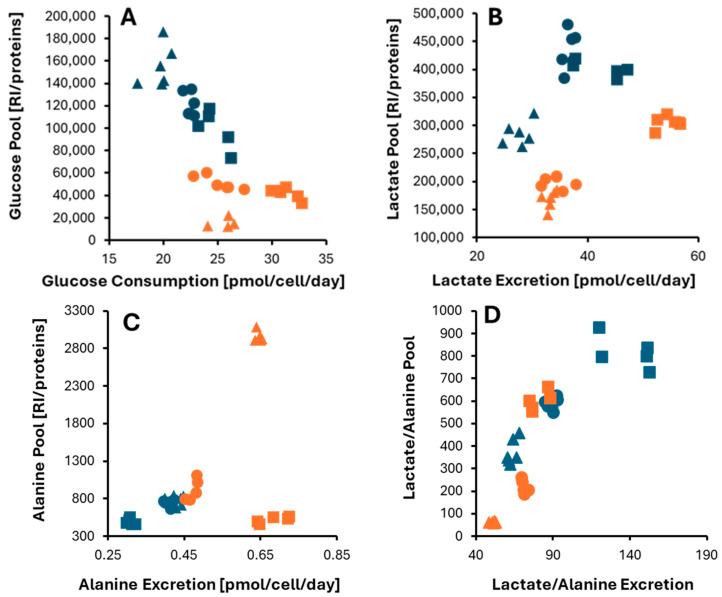
Correlation graphs of the internal pool and exchange rates for metabolites associated with the pyruvate metabolism: (**A**) glucose, (**B**) lactate, (**C**) alanine, and (**D**) the lactate/alanine ratio. Monolayer and spheroids data are colored in blue and orange, respectively. CCLP1, HUCCT1, and SG231 CCA cell lines are represented by circles, triangles, and squares.

Given its importance in defining the cell’s energy balance, we started by investigating the exchange rates in relation to the internal pools of three metabolites related to pyruvate metabolism: glucose, lactate, and alanine (Figure 8). LC-MS did not measure the pyruvate level inside the cells, so we could not include it in this approach. However, being primarily generated through glycolysis, the excretions and internal concentrations of lactate and alanine offer insights into how pyruvate is utilized within the cell.

The glucose internal level and exchange velocity differentiate 2D- vs. 3D-cultured cells (Figure 8A). Monolayers consume glucose slower and accumulate more within the cell than in 3D cultures. On the other hand, spheroids require larger quantities of glucose, accelerating its uptake, although it is metabolically consumed faster.

Cells in spheroids bear less internal lactate than those in monolayers (Figure 8B), which correlates with the lower glucose level already discussed. A higher lactate concentration yields a faster release when considering the two culture modes separately. For example, SG231 cells show a higher lactate content and excretion rate in both cultures than the other two cell lines. However, this is not true when comparing cells in 2D and 3D. Although monolayers’ internal level is higher, this is not reflected in a faster release. In particular, SG231 and HUCCT1 cells that show a higher lactate pool in 2D than in 3D cultures show a quicker release in spheroids. CCLP1 cells show lactate exchange rates similar in 2D and 3D cultures, although the lactate levels are higher in the former.

Alanine excretion is higher in spheroids than in monolayers (Figure 8C). Generally, a faster excretion correlates with a higher internal metabolite level, with one important exception: SG231 cells excreted more alanine in 3D than in 2D, although the internal concentration remained constant.

The cell lactate-to-alanine ratio (Lact/Ala) is an important indicator of pyruvate metabolism, as it reflects the redox state corresponding to the NADH/NAD+ ratio in different metabolic conditions [18]. Based on our research, this ratio appears to decrease in all three lines, as determined by internal pools and exchange rate quotients (Figure 8D).

In general, iCCA cell lines have been found to consume more glucose in 3D cultures, leading to a higher excretion of lactate and alanine. However, while alanine levels are higher within the cell, glucose and lactate pools become depleted compared to monolayer-cultured cells. To support this accelerated pyruvate metabolism in spheroids, pyruvate consumption was increased in two cell lines (CCLP1 and SG231).

#### 3.4.2. Glutathione Metabolism

**Figure 9:**
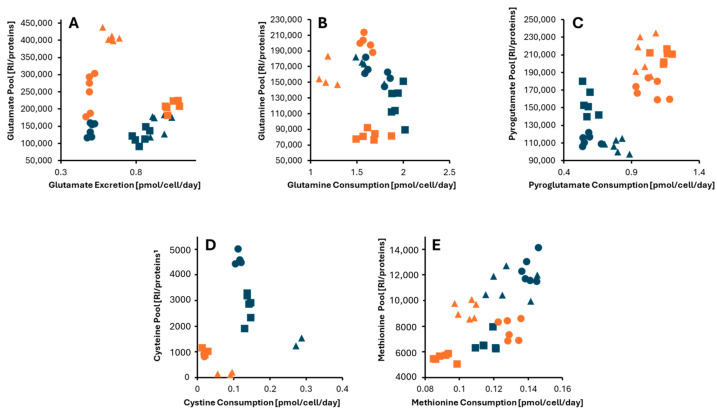
Correlation graphs of the internal pool and exchange rates for metabolites associated with the glutathione metabolism: (**A**) glutamate, (**B**) glutamine, (**C**) pyroglutamate, (**D**) cysteine, and (**E**) methionine. Monolayer and spheroids data are colored in blue and orange, respectively. CCLP1, HUCCT1, and SG231i CCA cell lines are presented by circles, triangles, and squares.

Glutathione is a significant antioxidant, and its level inside the cell can be used to monitor redox homeostasis in different cultures. To analyze this, we kept track of the exchange rates and levels of glutamate and cysteine, two glutathione precursors. We also monitored glutamine and pyroglutamate, which lead to glutamate, as well as methionine, another amino acid that is the source of sulfur atoms and a potential precursor of cysteine. Our results are presented in Figure 9.

While the exchange rates for the reduced (GSH) and oxidized (GSSG) forms of glutathione were not measured, LC-MS analysis revealed that their levels were consistently lower in 3D cultures than in 2D cultures. Interestingly, glutamate, a glutathione precursor, exhibited an opposing trend, with higher concentrations observed in spheroids (Figure 9A), although the exchange rates did not show a definitive pattern.

Glutamine is one of the primary precursors for glutamate synthesis. As shown in Figure 9B, the internal pool of glutamine is not significantly changed. Still, there is a tendency for a slower glutamine uptake by spheroids, in contrast to the increased internal glutamate pools. A possible alternative source is pyroglutamate, which shows both a faster uptake and an increased level inside the cells in 3D cultures (Figure 9C).

Cysteine and methionine, the two main sulfur sources of the cells in these conditions, show different behavior with respect to glutamate (Figure 9D,E). The first, a substrate for synthesizing glutathione, shows uptake rates and internal pools significantly decreased in 3D-cultured cells. Methionine shows similar trends (Figure 9E). The reduced uptake and internal levels align with the lower concentration of GSH and GSSG found in spheroids.

## 4. Discussion

Our research utilized NMR and LC-MS data to examine the exo- and endometabolomic profiles of three iCCA cell lines cultured as 2D monolayers and 3D spheroids. Our main goal was to understand how the culture method affects cell metabolism by analyzing metabolite exchange rates and internal concentrations. We discovered significant changes in the central carbon metabolism, with pyruvate and glutathione playing key roles.

Pyruvate is a vital molecule in cancer cells, supporting both energy production and biosynthesis for rapid cell growth [19]. The conversion of pyruvate into alanine and oxaloacetate also plays a role in cell signaling and the regulation of gene expression, impacting tumor growth and survival [20]. The primary biochemical pathway for pyruvate synthesis involves the process of glycolysis. Our research indicates that spheroids, unlike cells cultured in a monolayer, have a high glucose consumption rate and low internal glucose levels. The higher consumption of glucose can be linked to higher levels of GLUT-1, which have been seen in 3D spheroids compared to monolayers in two iCCA cell lines [21]. On the other hand, the lower concentration of glucose within the cells may result from elevated levels of key glycolytic enzymes, such as GAPDH and LDHA, which speed up glucose breakdown. This has been observed in spheroids for colorectal cancer and pancreatic ductal adenocarcinoma cell lines [22].

The difference in glucose metabolism between spheroids and monolayers mirrors that between cancer and healthy cells. This suggests that the spheroid microenvironment accentuates the metabolic differences in tumor cells, making their behavior more like that of cells in tumor tissues. Tumor cells overexpressing glucose transporters consume more glucose than normal cells, potentially serving as markers for cancer diagnosis and progression [23]. This heightened uptake is an adaptive strategy for survival and proliferation in environments with varying nutrient supply. Tumors generally have lower glucose concentrations than normal tissues due to increased consumption [24]. Unlike normal cells, tumor cells have lower internal glucose stores and quickly absorb available glucose to create reserves against future scarcity [25], responding with increased metabolic activity. Spheroids have a significant glucose gradient from the surface to the inner layers, triggering different adaptation strategies due to varying glucose levels [26].

In addition, we observed increased lactate excretion in spheroids with respect to 2D-cultured cells, as already observed in dermal fibroblasts cultured in the two modes [27]. It is known that low-nutrient conditions, such as those found in the inner layers of spheroids, drive cancer cells to utilize glycolysis to produce ATP through the ROS/AMPK-dependent activation of pyruvate dehydrogenase kinase [28]. Our data on the glucose uptake, internal pool, and high lactate excretion in spheroids are compatible with this glycolytic shift.

Although alanine and lactate were excreted more in spheroids, the Lact/Ala ratios of the excretion rates and the internal pools of both metabolites diminished for all lines. This ratio is commonly utilized as an indirect gauge of cellular redox states, especially when NADH/NAD+ ratios are challenging to measure directly [18]. This link can be attributed to the enzymatic processes involved in lactate and alanine production. Lactate dehydrogenase generates lactate from pyruvate while consuming NADH, whereas alanine is produced through a transamination reaction that does not affect NADH/NAD+ levels. This ratio was lower in 3D cultures than in 2D cultures of murine breast cancer cells line 4T1 [29]. The lower ratio in spheroids can have different interpretations depending on the oxygen availability. Cells at the periphery of spheroids might have better access to oxygen, thus favoring oxidative phosphorylation over glycolysis for ATP production. This process consumes NADH, producing NAD+, and could explain lower NADH/NAD+ ratios. In areas of the spheroid with less oxygen availability, cells might rely on glycolysis for ATP production, which requires the conversion of NAD+ to NADH. For glycolysis to proceed, NADH must be recycled back to NAD+, usually by converting pyruvate to lactate when oxygen is limited. This recycling helps maintain a lower NADH/NAD+ ratio.

The glutathione metabolism is directly related to the redox state of cells. We observed a marked reduction in the internal pool of GSH and GSSG and their precursors, like cysteine and cysteinylglycine, coupled with a decreased cystine uptake. The same effect was observed for two CCA cell lines in 3D multicellular spheroid models [21]. A decrease in GSH levels can impair the cell’s ability to combat oxidative damage, leading to increased oxidative stress [30]. This altered redox balance in tumor cells can induce genetic mutations, promote genomic instability, and potentially lead to more aggressive cancer phenotypes [31]. Lower levels of GSH in 3D-cultured cells might initially seem detrimental in terms of reduced antioxidant capacity; however, it can also lead to the selection of more resilient tumor cells that adapt to high-oxidative-stress environments. These cells may exhibit enhanced resistance to chemotherapy and radiation therapy, which often rely on generating ROS to kill cancer cells. This may explain why spheroids usually better recapitulate the therapy response observed in tumors than monolayer-cultured cells [32,33].

Glutamate is involved in GSH synthesis and has various biochemical functions. The increase in glutamate in spheroids is partially due to a decreased GSH concentration. There is also a decrease in glutamine influx, suggesting a reduced reliance on this metabolite for cell function in spheroids, as already observed in the case of MGH-U1 bladder cancer cells [34]. Pyroglutamate may serve as an alternative source for glutamate biosynthesis, similar to what was observed in nervous tissue [35]. The increase in the pyroglutamate consumption and internal pool may be related to the rise in internal glutamate in spheroids.

In 2D cultures, cells are spread across a flat surface, allowing for direct and uniform access to nutrients and oxygen in the culture media. This uniform exposure facilitates a relatively homogeneous condition where all cells equally partake in nutrient uptake and waste exchange with the surrounding medium [36]. This homogeneity ensures that the observed nutrient exchange rates represent the entire cell population. Conversely, 3D spheroids present a more complex structure, where only the outer layer of cells directly access the culture medium [37]. When measuring nutrient exchange rates in 3D cultures, the main contribution comes from the outermost cells in direct contact with the medium. The exometabolomics profile of the spheroids is predominantly influenced by the superficial spheroid layer that has comparable access to nutrients and oxygen as 2D-cultured cells. This similarity diminishes the impact of the culture type in the exometabolomics profile, increasing the effect of the differences among cell lines. This includes, for example, the significantly reduced consumption of BCAAs and essential amino acids by SG231 cells, a feature not shared by the other two cell lines.

On the other hand, while monolayers continue to be homogeneously represented in terms of internal cell contents, the metabolite levels inside the cells of the entire spheroid represent a weighted average of the different populations forming the various layers. This amplifies the differences observed between 2D- and 3D-cultured cells, explaining why the type of culture contributes mostly to the differences in the internal pools. As a result, twenty-eight metabolite levels were altered in the same way in all three cell lines.

Despite these facts, we could still identify the variations in the exometabolomic profiles associated with the two culture types using OPLS-DA models. Our findings indicate that their metabolisms differ significantly, although the cells cultured in 2D and those in the outer layer of the spheroid have access to adequate nutrients and oxygen. The most significant differences were observed in the alanine, aspartate and glutamate, glyoxylate and dicarboxylate, and glutathione pathways. A similar metabolic impact on these metabolisms was measured by employing nanocapillary-based electrospray ionization mass spectrometry [38]. Even if cells found in the outer layer of a spheroid or tissue divide, the conditions they encounter are different from those in cell monolayers. In the latter case, cells divide and grow rapidly until they reach the confluence condition, where cell-cell contacts occur. However, the surface cells of spheroids interact with other cells even during division, creating a diverse microenvironment that plays a crucial role in metabolic reprogramming. These contacts and the diverse microenvironment may be determinants in explaining the metabolic reprogramming that we observed. There is ample evidence supporting the fundamental role of cell-cell interaction in differentiating tumor cells’ metabolic behavior between 2D and 3D cultures [39,40].

Compared to the homogeneity of monolayers, the diversity of the population in spheroids influences the comparison of endometabolomic profiles. 3D-cultured cells show a significant metabolic stratification, mainly caused by the gradients of oxygen, nutrients, and other factors that result from the limited diffusion into and out of the spheroid. For example, the drift towards more active glycolysis that emerges from our study’s analysis of the endometabolic data is partially influenced by the presence of a glycolytic core generated in the absence of oxygen in the interior of the spheroids. However, under certain conditions, some spheroids can show an “inverted” oxygenated core and, consequently, a region with increased glycolytic activity near the spheroid’s periphery [41]. These results show that the static model in which the outer cells with access to oxygen use mostly OXPHOS and the inner core glycolysis does not represent the true dynamicity of the metabolic heterogeneity of 3D cultures.

We have also observed a decrease in the level of GSH inside cells cultured in 3D. This alteration can be attributed to an inner core with excess ROS inside the cell. This excess drastically decreases the concentration of this metabolite and initiates the cellular apoptotic program. However, recent studies have shown that GSH can be found at lower levels than in monolayer cells, even at the spheroid surface [38]. This indicates that the increased oxidative stress to which cells are exposed in 3D cultures is not a condition exclusive to the inner core.

In summary, our study showed metabolic differences between 2D and 3D cultures, emphasizing the complex interplay between exchange rates and internal pools. Combining datasets revealed significant alterations in central carbon metabolism, affecting glycolytic activity and cellular redox balance. Our results suggest that the differences in cell metabolism between spheroids and monolayers are similar to those observed between tumor and healthy cells [23], indicating that 3D cultures better mimic the microenvironment of cancerous tissues.

A crucial advantage of 3D tumor models is their ability to recapitulate the heterogeneity of in vivo tumors. This heterogeneity, which includes varying cell populations and microenvironments, plays a pivotal role in enhancing drug resistance—a phenomenon frequently observed in cancer [42,43]. Unlike monolayers, 3D spheroids allow for the development of drug efflux mechanisms and support the survival of resistant subpopulations, such as “side population” cells, known for their ability to evade chemotherapeutic agents through high-efflux activity [44]. Although not fully captured in this analysis, recognizing these dynamics in 3D models is essential for refining future therapeutic approaches and improving the relevance of in vitro systems in mimicking the complex drug resistance seen in cholangiocarcinoma and other cancers.

The main limitation of this type of cell culture is the lack of a complex vascular system that provides oxygenation, nutrition, and waste removal in living tissues, even in internal areas. Cells cultured in 3D rely solely on diffusion for these functions, leading to significant differences. This may result in exacerbated nutrient deprivation or hypoxic conditions. While this may not be an important issue for small spheroids, larger spheroids face challenges in replicating vascularized systems. Some solutions have been proposed to address this [45,46,47]. Establishing a cellular model that closely mimics in vivo tissue can facilitate examining metabolism and evaluating responses to various therapies. This, in turn, can expedite the discovery of novel approaches to fighting cancer.

## Data Availability

The corresponding authors can provide data on request.

## Supplementary Materials

The following supporting information can be downloaded at: https://www.mdpi.com/article/10.3390/cells13181536/s1. Figure S1: Representative images of 2D and 3D culture of CCA cell lines. Refs. [48,49,50,51] are cited in the Supplementary Materials file.
