# Supplementary material for: 3D Modeling: Insights into the Metabolic Reprogramming of Cholangiocarcinoma Cells
Source: Cells. 2024 Sep 13;13(18):1536. doi: 10.3390/cells13181536 (PMC11430555; doi:10.3390/cells13181536)
Supplement: Supplementary file 1 [file cells-13-01536-s001.zip › cells-3202049-supplementary.pdf]

Supplementary Materials

Cell Culture

Cholangiocarcinoma cell lines CCLP1and SG231 were routinely grown in DMEM High Glucose medium supplemented with 10% fetal bovine serum (FBS), 2 mM glutamine, 1% HEPES, 100 U/mL penicillin, and 100 mg/mL streptomycin at 37°C in a humidified atmosphere of 5% CO_2. Cholangiocarcinoma cell line HUCCT1 was routinely grown in RPMI-1640 medium supplemented with 10% fetal bovine serum (FBS), 2 mM glutamine, 1% HEPES, 100 U/mL penicillin, and 100 mg/mL streptomycin at 37°C in a humidified atmosphere of 5% CO_2. Cells were passaged using trypsin-ethylenediaminetetraacetic acid (EDTA).

Sphere formation was performed in 3D experimental medium DMEM High glucose (Gibco™-Thermo Fisher Scientific): Ham's F-12 Nutrient Mix ratio 1:1, B27, 2 mM glutamine, 20 ng/mL EGF, 20 ng/mL bFGF, Matrigel Corning 354234 2.5%. In a typical experiment, 1.0 x 10^4 cells/well were plated in CellCarrier Spheroid ULA 96-well Microplates with ultra-low attachment surface; after cell seeding, the plates were centrifuged to promote cell aggregation and incubated at 37°C and 5% CO_2 [1]. After five days of growth, the medium was collected, and the pellet was saved for endometabolomics experiments. Total live cell count was performed on day 0 and day 5 of the analysis after trypsinization with the trypan blue exclusion method to evaluate viability. Sphere morphology was monitored through imaging acquisitions after the 72 h and 120 h experiments, using Operetta CLS™ in brightfield fluorescence microscopy with 5× magnification [2]. We analyzed sphere formation using the Harmony software. Only well-formed spheroids of the same size were chosen for analysis.

For the two-dimensional experiment, cells were grown in adhesion in 6-well plates in 2 mL in the same 3D experimental medium used for spheroids. In a typical experiment, the cells were plated, and after one day of growth, the medium was changed, and any unadhered cells were removed. At this point, the cells were incubated for 48 h at 37°C and 5% CO_2. After two days of growth, the medium was collected, and the pellet was saved for endometabolomics experiments. To measure the number of cells and evaluate their viability at the initial and final time of the experiment, replicates obtained in parallel experiments were used for the cell count after Hoechst staining and quantitative imaging using Operetta CLS™ system.

We obtained two biological replicates for the two-dimensional and three-dimensional experiments, each composed of three technical replicates, for each cell line after two days of culture for 2D and five days for 3D experiments.

Samples of 3D experimental medium (without cells) were also collected under the same experimental conditions to be used as a blank for the experiments. Then, the collected samples were stored at −80°C until the NMR analysis.

NMR-based exometabolomics analysis

Samples for NMR Analysis

The medium from each cell culture was collected, centrifuged to remove cell residues, and immediately frozen at −80°C. Before the NMR analysis, each sample was thawed at room temperature. The sample was subjected to a deproteinization process by ultrafiltration to remove eventual proteins in the medium. To this purpose, 500 μL of the sample was inserted in an Amicon Ultra 0.5 filter membrane with a cut-off equal to 3 KDa (previously cleared of glycerol) and subjected to centrifugation at 13,800×g at 4°C for 90 min. Subsequently, 400 μL of the filtrate was placed into an Eppendorf and added to 150 μL of phosphate buffer (250 mM KH_2PO_4/K_2HPO_4, pH 7.4) containing 2.4 mM 3-(trimethylsilyl)-2,2,3,3-tetradeutero-propionic acid (TSP-d_4), 10% D_2O, 2% NaN_3, and 50 μL of Imidazole (used as internal pH calibrator). The final solution was transferred to a 5 mm NMR tube for the acquisition.

Spectra acquisition

Acquisition of ^{1}H-NMR spectra was performed using a Bruker Avance 700 MHz spectrometer equipped with SampleXpress Lite autosampler and Topspin software (version 2.1). For each sample, a ^{1}H-NMR spectrum was acquired with a noesypr1d sequence at 298 K, 15.9 ppm spectral window, 256 scans, four dummy scans, 2s relaxation delay, and 3s acquisition time. The total duration of each experiment was 22 min.

Spectral analysis

The assignment and the quantification of the concentration of the different metabolites were performed using Chenomx NMR Suite 8.5 (Chenomx Inc., Edmonton, AB, Canada). To this end, a manual deconvolution of the various signals was performed using the spectra database contained in this software. The spectra were processed using a 0.5 Hz line broadening followed by manual phase and baseline corrections. TSP was set as internal standard, and 33 metabolites were quantified in almost all samples.

Measurement of the metabolite exchange rates

To properly evaluate the variations in metabolite concentrations in the extracellular medium due to the intrinsic metabolism, we assessed the metabolite exchange rates with the culture medium by calculating the ratio between the metabolite variations and the area under the growth curve of cells [3].

The variation in mass of all quantified metabolites, $\Delta[m]_i^k$, was calculated using equation [1]:

$$\Delta[m]_i^k = ([M]_i^k - [M]_i^0) \cdot V \qquad [1]$$

where $[M]_i^k$ refers to the concentration of the i-metabolite in the k sample and $[M]_i^0$ to the concentration in the fresh medium cultured in the same condition and V the total medium volume. The mass variation obtained in this way is then normalized by the area under the growth curve [4]. For the two-dimensional culture model, the growth was considered

exponential, leading to equation [2] for the area (A_c) by integration between the starting (t_1) and final (t_2) times of culture:

$$A_c = \int_{t_1}^{t_2} n_0 e^{k_c t}\, dt = \frac{n_0}{\ln(n_2) - \ln(n_0)} \left(\frac{n_2}{n_0} - 1\right) \qquad [2]$$

Where k_c is the exponential rate, n_0 the initial number of cells and n_2 after two days. On the other hand, we observed a much lower cell number variation for the 3D experiment, leading to a linear growth curve approximation and the use of equation [3] that accounts for cases in which the cell number increases or decreases after five days:

$$A_c = \left\{\frac{t \times [MAX(n_0; n_5) - MIN(n_0; n_5)]}{2}\right\} + \{\, t \times [\, MIN(n_0; n_5)]\} \qquad [3]$$

where t is the time of culture and n_0 the initial number of cells and n_5 after five days.

Finally, metabolite exchange rates (ER) in [pmol cell^{-1} day^{-1}] were calculated using equation [4]:

$$ER_i^k = \frac{\Delta[m]_i^k}{A_c} \qquad [4]$$

Applying equation [4] yields negative exchange rates for consumption and positive exchange rates for excretion. Of the 33 analyzed metabolites, seven were excreted across all lines and conditions, twenty-one were consumed, and five demonstrated mixed behavior. Two distinct variables were formulated for excretion and consumption for the metabolites that exhibited mixed behavior: acetate, asparagine, aspartate, proline, and sarcosine, resulting in a total of 38 exchange rates. The resulting velocities were subsequently transformed by taking their absolute value and suitably classified for further examination.

LC-MS-based endometabolomics analysis

Metabolite extraction for internal pool analysis

For 2D cell culture, cells in a six well-plate were quickly rinsed with NaCl 0.9% and quenched with 500 µl ice-cold 70:30 acetonitrile:water. Plates were placed at – 80°C for 10 min; then, cells were collected by scraping and sonicated for 5 s for five pulses at 70% power twice. For 3D cell culture, 48 spheroids were collected from a 96-well plate for each sample and centrifuged at 600 rpm for 5 mins at 4°C. Pellets were washed with 1 ml of NaCl 0.9%, centrifuged as above, and resuspended in 500 µl ice-cold 70:30 acetonitrile:water. Samples were placed at – 80°C for 10 min and then sonicated for 5 s for five pulses at 70% power twice. At this point, for both 2D and 3D experiments, samples were centrifuged at 12000g for 10 min, and supernatants were collected in a glass insert and dried in a centrifugal vacuum

concentrator (Concentrator plus/Vacufuge plus, Eppendorf) at 30°C for about 2.5 h. Samples were then resuspended with 150 µl H_2O before analyses.

UHPLC-QTOF analysis

LC separation was performed using an Agilent 1290 Infinity UHPLC system and an InfintyLab Poroshell 120 PFP column (2.1 × 100 mm, 2.7 µm; Agilent Technologies). Mobile phase A was water with 0.1% formic acid. Mobile phase B was acetonitrile with 0.1% formic acid. The injection volume was 10 µL, and LC gradient conditions were: 0 min: 100% A; 2 min: 100% A; 4 min: 99% A; 10 min: 98% A; 11 min: 70% A; 15 min: 70% A; 16 min: 100% A with 2 min of post-run. The flow rate was 0.2 ml/ min, and the column temperature was 35 ◦C. MS detection was performed using an Agilent 6550 iFunnel Q-TOF mass spectrometer with a Dual JetStream source operating in negative ionization mode. MS parameters were gas temp: 285 ◦C; gas flow: 14 l/min; nebulizer pressure: 45 psig; sheath gas temp: 330 ◦C; sheath gas flow: 12 l/min; VCap: 3700 V; Fragmentor: 175 V; Skimmer: 65 V; Octopole RF: 750 V. Active reference mass correction was done through a second nebulizer using masses with m/z: 112.9855 and 1033.9881. Data were acquired from m/z 60–1050. Data analysis and isotopic natural abundance correction were performed using MassHunter Profinder (version 10.0.2). Data preprocessing was performed using the Batch Targeted Feature Extraction algorithm and Agile 2 algorithm. This software assigned identities to metabolites by searching against an in-house compound database built with Agilent PCDL Manager (version B.08.00) based on the metabolite formula and its corresponding retention time with a score > 75. Peak areas obtained were normalized for protein content for each sample.

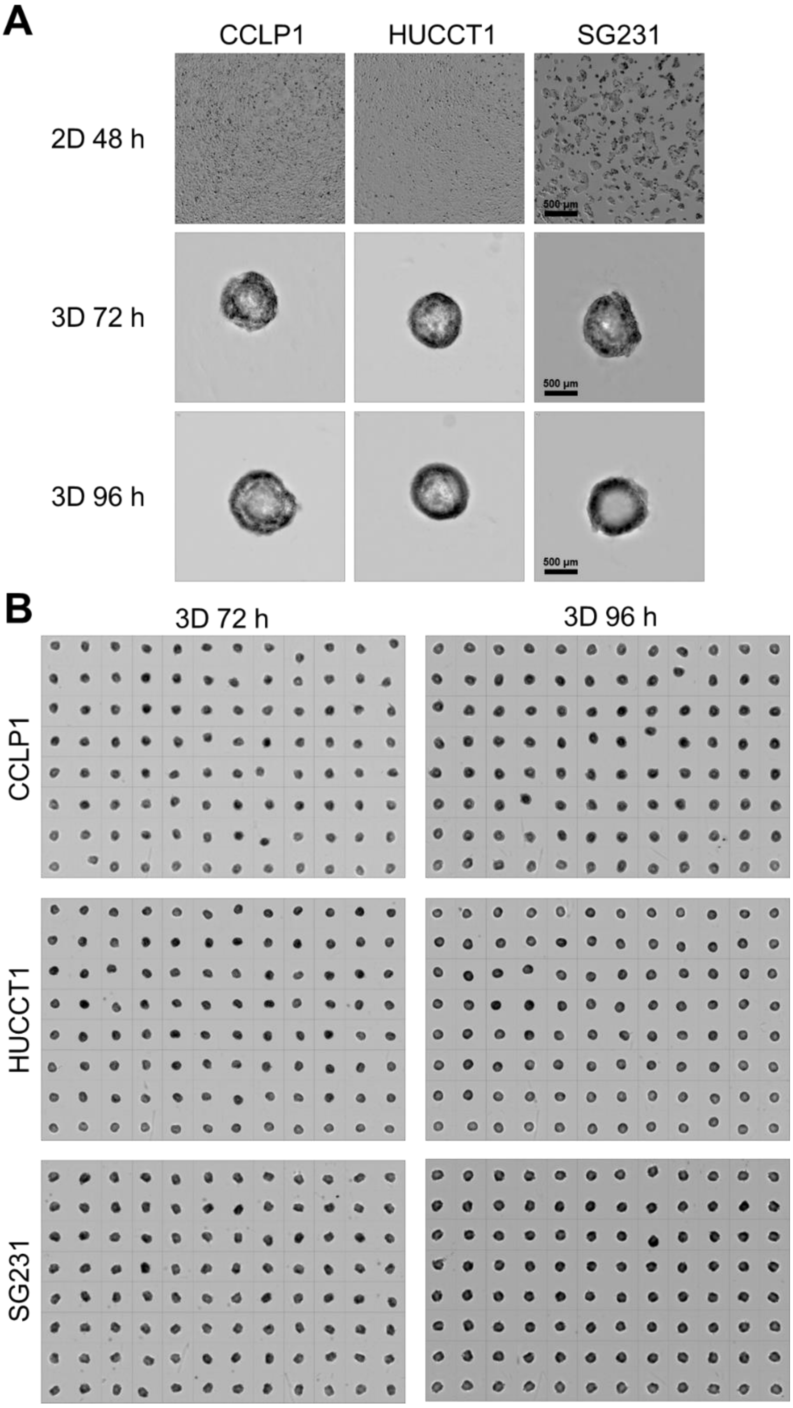

Figure S1. *Representative images of 2D and 3D culture of CCA cell lines. A) Images were acquired by brightfield (5x magnification) microscopy with Operetta CLSTM at 48h for 2D culture and 72h and 96h for 3D culture; B) overview of the plate for spheroid formation at 72h and 96h of the three CCA cell lines for metabolomics analysis.*

References

1. Campioni, G.; Pasquale, V.; Busti, S.; Ducci, G.; Sacco, E.; Vanoni, M. An Optimized Workflow for the Analysis of Metabolic Fluxes in Cancer Spheroids Using Seahorse Technology. *Cells* **2022**, *11*, 866, doi:10.3390/CELLS11050866/S1.

2. Pasquale, V.; Ducci, G.; Campioni, G.; Ventrici, A.; Assalini, C.; Busti, S.; Vanoni, M.; Vago, R.; Sacco, E. Profiling and Targeting of Energy and Redox Metabolism in Grade 2 Bladder Cancer Cells with Different Invasiveness Properties. *Cells* **2020**, *9*, 1–26, doi:10.3390/CELLS9122669.

3. Petrella, G.; Corsi, F.; Ciufolini, G.; Germini, S.; Capradossi, F.; Pelliccia, A.; Torino, F.; Ghibelli, L.; Cicero, D.O. Metabolic Reprogramming of Castration-Resistant Prostate Cancer Cells as a Response to Chemotherapy. *Metabolites* **2023**, *13*, doi:10.3390/metabo13010065.

4. Jain, M.; Nilsson, R.; Sharma, S.; Madhusudhan, N.; Kitami, T.; Souza, A.L.; Kafri, R.; Kirschner, M.W.; Clish, C.B.; Mootha, V.K. Metabolite Profiling Identifies a Key Role for Glycine in Rapid Cancer Cell Proliferation. *Science* **2012**, *336*, 1040–1044, doi:10.1126/SCIENCE.1218595.
